# Supplementary material for: Impact of Reduced Saliva Production on Intestinal Integrity and Microbiome Alterations: A Sialoadenectomy Mouse Model Study
Source: Int J Mol Sci. 2024 Nov 20;25(22):12455. doi: 10.3390/ijms252212455 (PMC11594800; doi:10.3390/ijms252212455)
Supplement: Supplementary file 1 [file ijms-25-12455-s001.zip › ijms-3301476-supplementary.pdf]

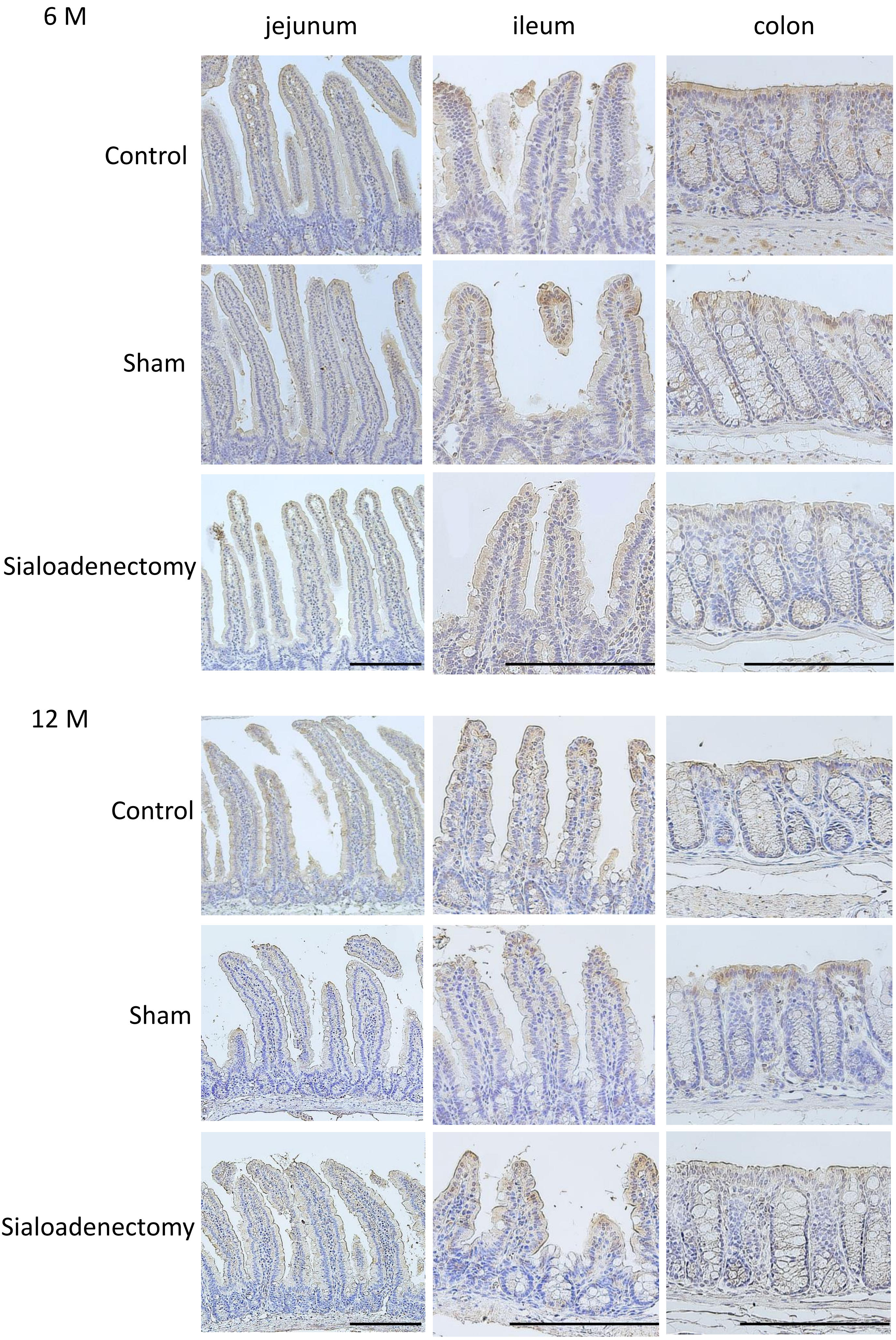

Supplementary Figure S1: Immunohistochemical analysis of EGF expression in the sialoadenectomy, sham, and control groups. Representative results for each group are shown. At 6 and 12 months after surgery, the level of EGF expression did not show apparent differences across the three groups. Scale bar: 200 μm.

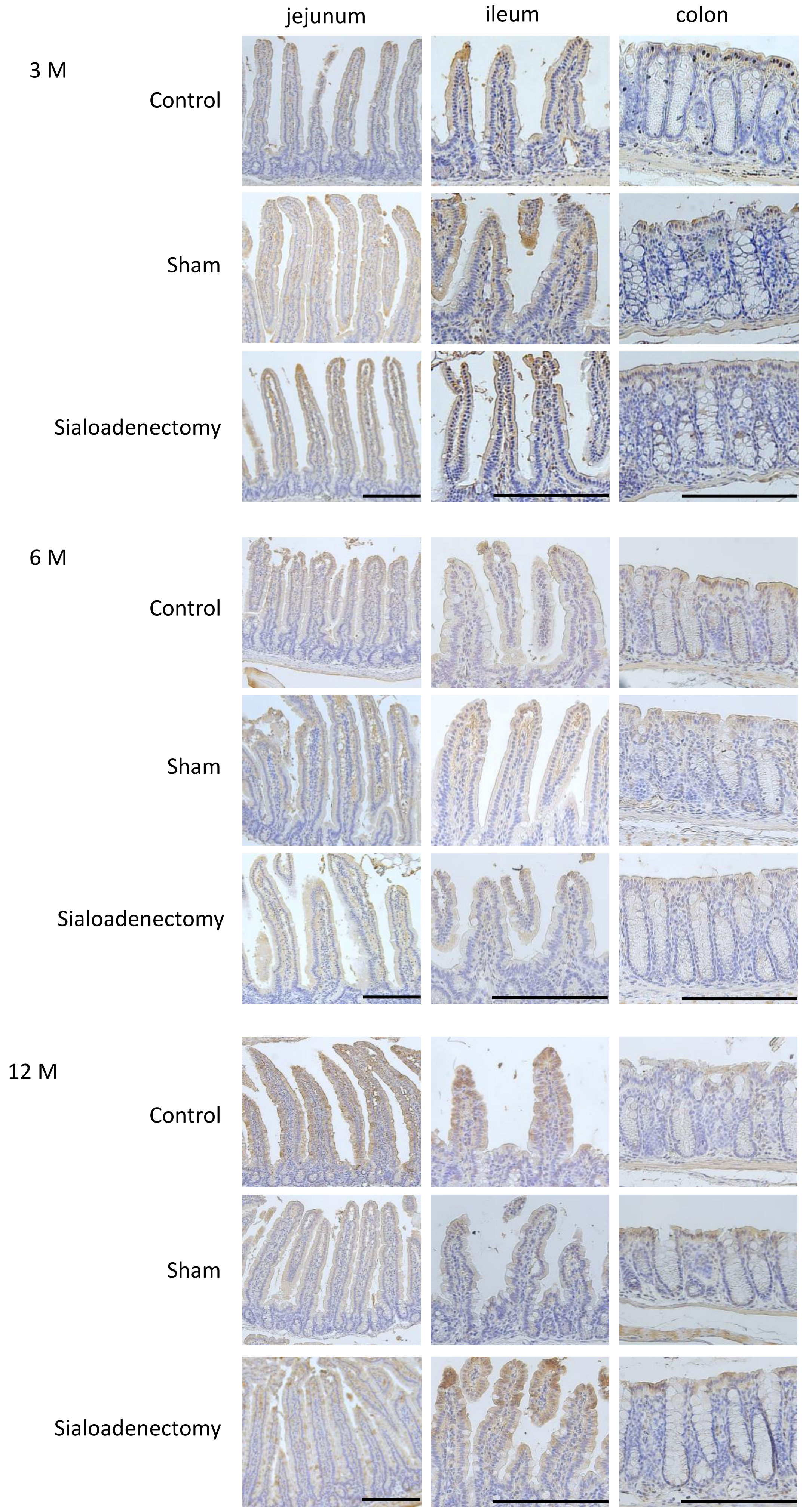

Supplementary Figure S2: Immunohistochemical analysis of VEGF expression in the control, sham, and sialoadenectomy groups. Representative results for each group are shown. Throughout the 12-month experimental period, VEGF expression was strongly positive in the villi of the jejunum and ileum and colonocytes in the colon. Scale bar: 200 μm.

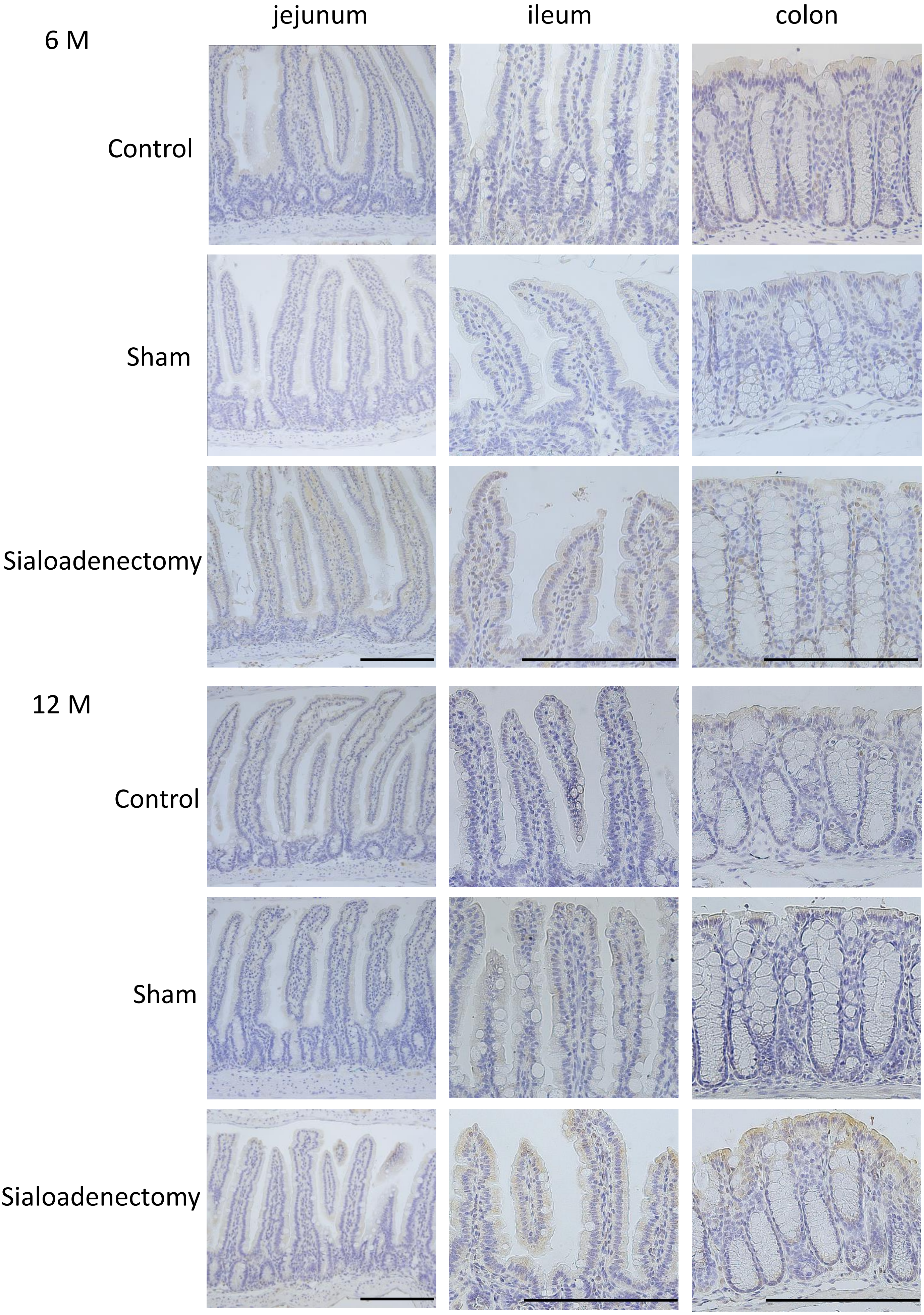

Supplementary Figure S3: Immunohistochemical analysis of PARP expression in the control, sham, and sialoadenectomy groups. Representative results for each group are shown. At 6 and 12 months after surgery, the mucosal epithelium surface in the control and sham groups showed slight PARP positivity. In the sialoadenectomy group, PARP expression was decreased at 6 and 12 months after surgery compared with 3 months after surgery but remained more strongly positive than the other two groups. Scale bar: 200 μm.

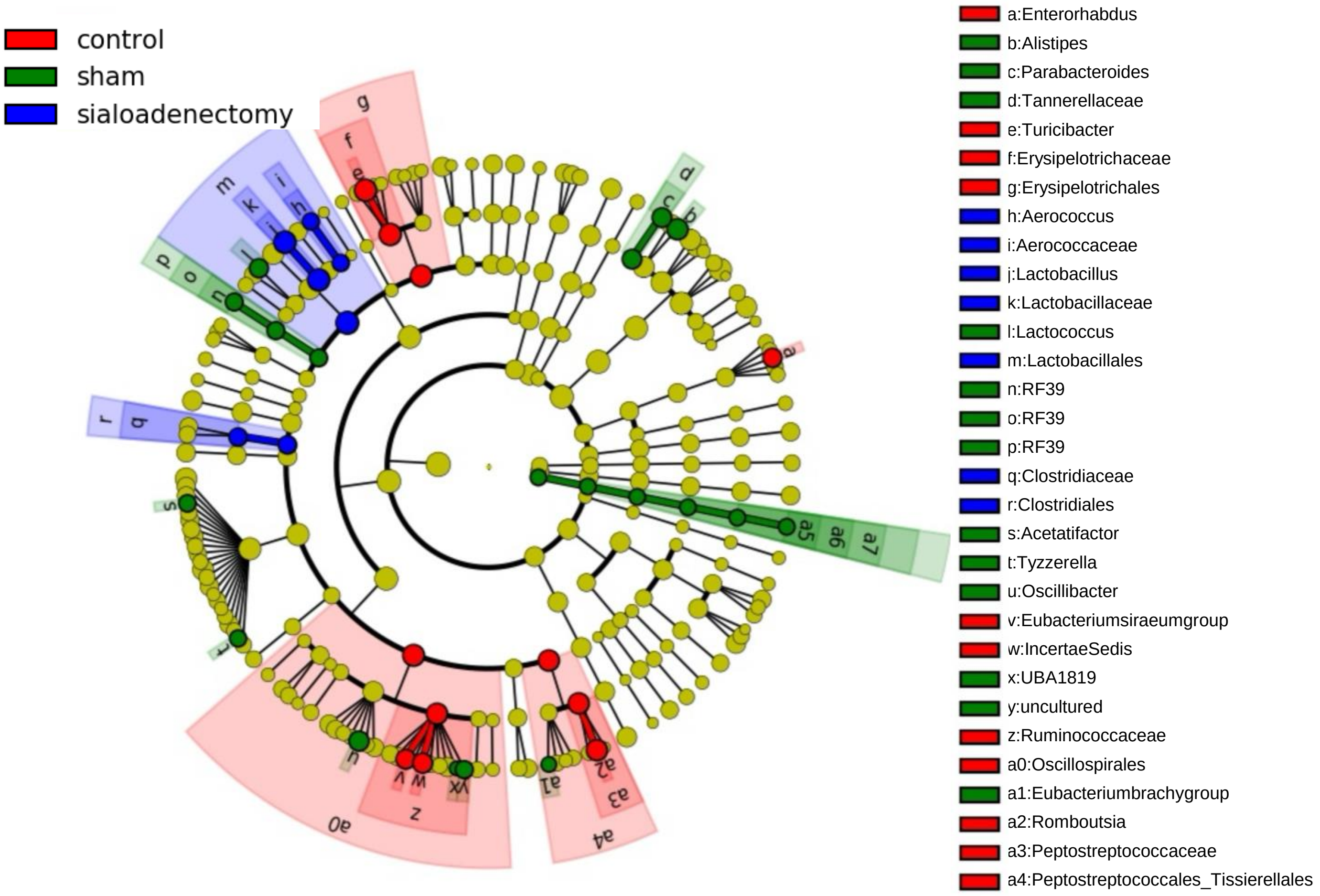

Supplementary Figure S4:High-resolution cladogram plotted from the LEfSe analysis at 3 months after surgery

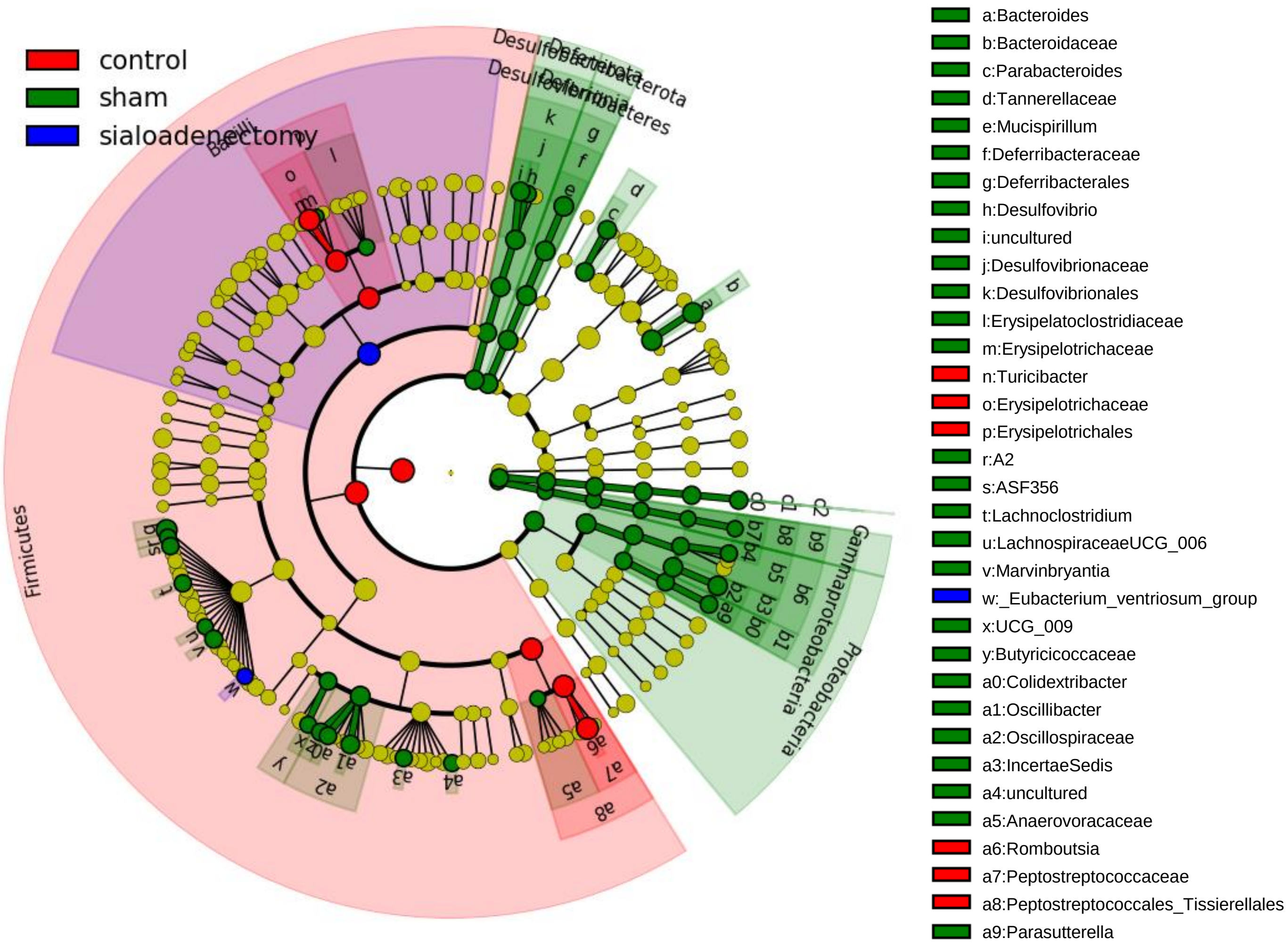

Supplementary Figure S5:High-resolution cladogram plotted from the LEfSe analysis at 6 months after surgery

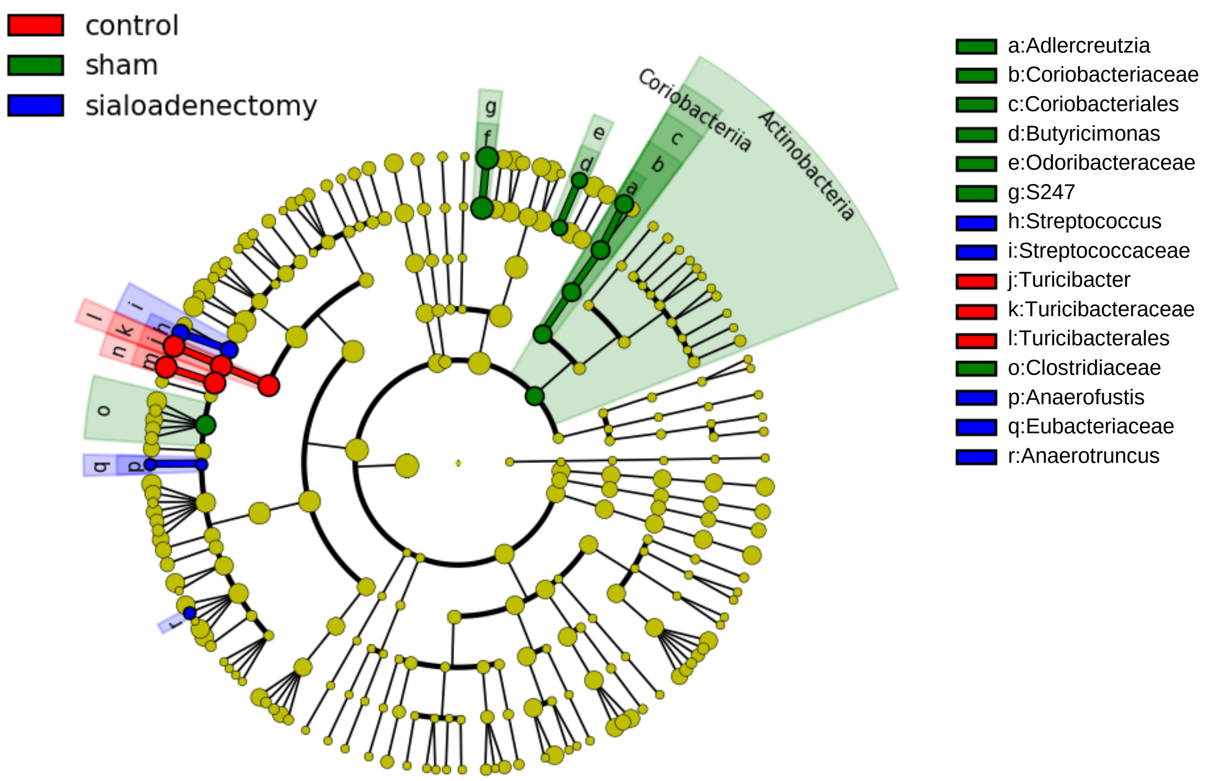

Supplementary Figure S6:High-resolution cladogram plotted from the LEfSe analysis at 12 months after surgery

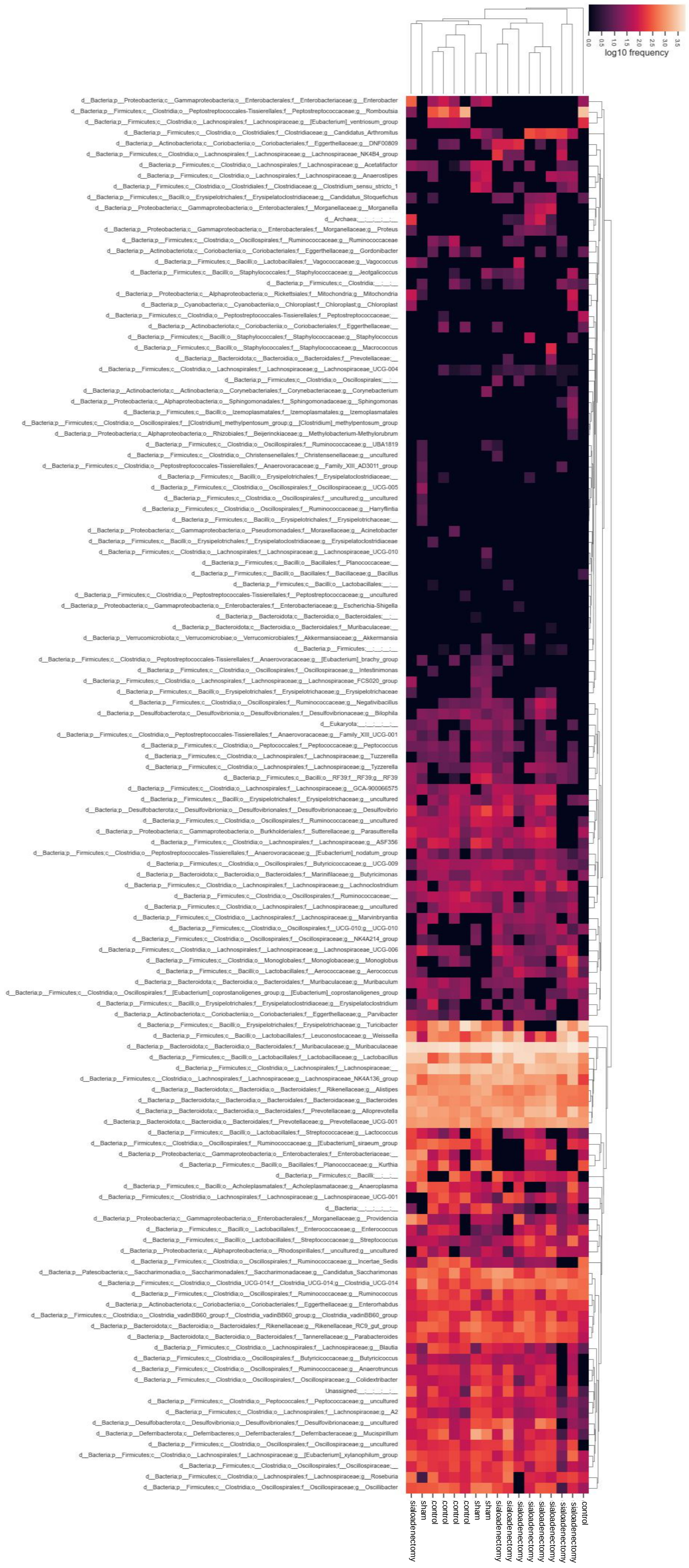

Supplementary Figure S7: Whole heatmap analysis at 3 months after surgery

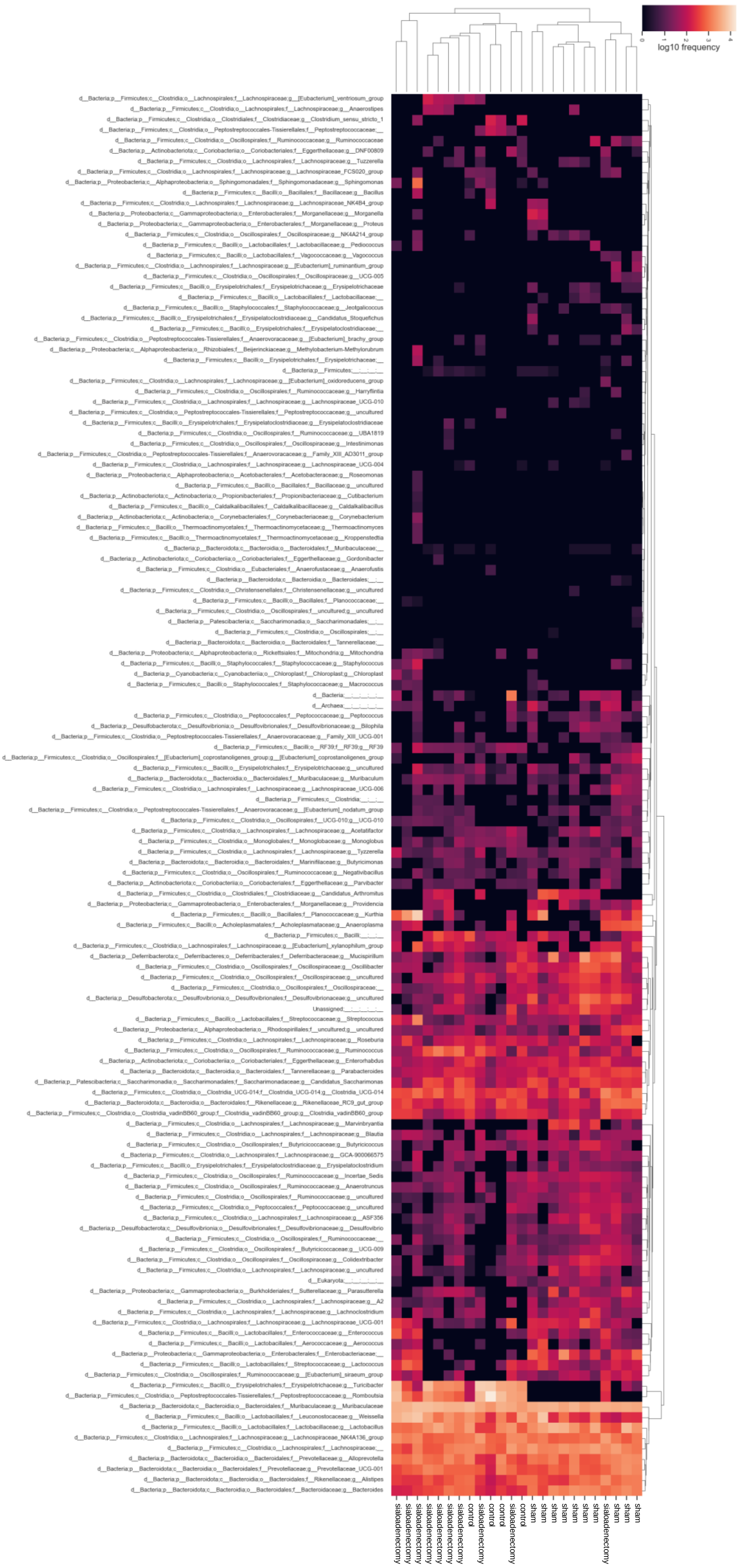

Supplementary Figure S8: Whole heatmap analysis at 6 months after surgery

Table S1 Relative abundance of gut microbiota more than 5%.

	level	control	sham	sialoadenectomy
3M	phylum	2	2	2
	class	3	3	3
	order	6	4	3
	family	7	6	5
	genus	4	7	7
	species	5	5	7
6M	phylum	2	2	2
	class	3	3	3
	order	5	4	5
	family	7	6	7
	genus	5	5	6
	species	5	5	6
12M	phylum	2	2	2
	class	3	3	3
	order	4	3	4
	family	5	4	6
	genus	5	4	6
	species	4	4	5

Table S2 relative abundance of gut microbiota more than 0.1%.

	level	control	sham	sialoadenectomy
3M	phylum	9	9	9
	class	11	11	11
	order	21	23	23
	family	32	33	34
	genus	58	60	59
	species	69	76	76
6M	phylum	7	10	9
	class	8	12	11
	order	17	23	23
	family	25	36	34
	genus	36	61	55
	species	45	82	71
12M	phylum	7	7	8
	class	10	11	12
	order	11	12	12
	family	23	27	27
	genus	29	34	33
	species	30	37	34
